# MicroRNA-376c-3p Facilitates Human Hepatocellular Carcinoma Progression via Repressing AT-Rich Interaction Domain 2: Erratum

**DOI:** 10.7150/jca.76884

**Published:** 2022-08-19

**Authors:** Yuan Wang, Weiping Chang, Wanli Chang, Xiaowei Chang, Song Zhai, Guoying Pan, Shuangsuo Dang

**Affiliations:** 1 Department of Infectious Diseases, the Second Affiliated Hospital of Xi'an Jiaotong University, 157 Xiwu Road, Xi'an 710004, China; 2 Department of General Surgery, the First Affiliated Hospital of Xi'an Medical University, 48 Fenghao West Road, Xi'an 710077, China

We regret that the original version of our paper unfortunately contained one incorrect representative image. The wrong image was placed in the miR-376c-3p inhibitors + NT siRNA group in Figure 6C when choosing a representative image from the countless image data. The correct version of the Figure 6C appears below. The authors confirm that the corrections made in this erratum do not affect the original conclusions. All the authors of the paper have agreed to this correction. The authors apologize for any inconvenience that the errors may have caused.

## Figures and Tables

**Figure 6:**
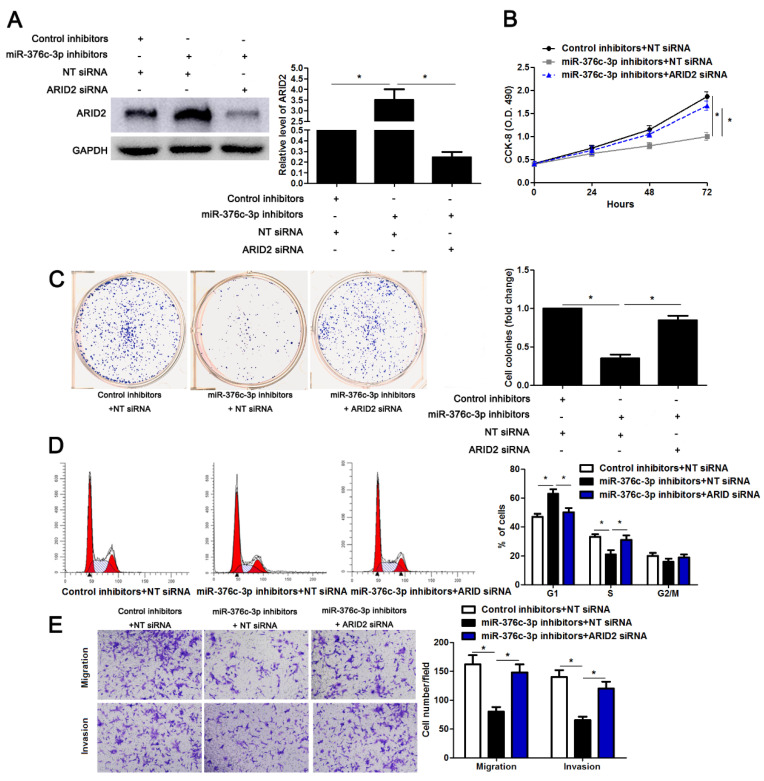
**ARID2 silencing abolishes the effect of miR-376c-3p knockdown in HCC cells.** (A) miR-376c-3p inhibitors and ARID2 siRNA were co-transfected into Hep3B cells. The protein level of ARID2 was determined using immunoblotting. n=three independent repeats, *P<0.05 by ANOVA. (B) miR-376c-3p knockdown suppressed proliferation of Hep3B cells, and ARID2 silencing subsequently promoted cell proliferation. n=three independent repeats, *P<0.05 by ANOVA. (C) ARID2 silencing increased the number of cell colonies in miR-376c-3p silenced Hep3B cells. n=three independent repeats, *P<0.05 by ANOVA. (D) miR-376c-3p knockdown led to G1 arrest, and ARID2 silencing subsequently promoted cell cycle progression in Hep3B cells. n=three independent repeats, *P<0.05 by ANOVA. (E) The migration and invasion capacities were repressed by miR-376c-3p knockdown, and accordingly reversed by ARID2 silencing in Hep3B cells. n= five filed of three independent repeats, *P<0.05 by ANOVA.

